# Transcriptome Analysis Reveals AI-2 Relevant Genes of Multi-Drug Resistant *Klebsiella pneumoniae* in Response to Eugenol at Sub-MIC

**DOI:** 10.3389/fmicb.2019.01159

**Published:** 2019-05-28

**Authors:** Yi-ming Wang, Wen-long Dong, Kokou Ayefounin Odah, Ling-cong Kong, Hong-xia Ma

**Affiliations:** College of Animal Science and Technology, Jilin Agricultural University, Changchun, China

**Keywords:** eugenol, *K. pneumoniae*, quorum sensing, transcriptome analysis, AI-2 synthesis, inhibitory effect

## Abstract

Eugenol, the major active essential oil component of clove, was reported to possess QS (quorum sensing) inhibitory activity. A previous study found that eugenol could bind to quorum sensing receptors of *Pseudomonas aeruginosa* and down-regulate the expression of *Streptococcus mutans* virulence genes at sub-MIC (minimum inhibitory concentration) without affecting the bacterial growth. However, the alterations of QS signal molecules at transcription levels was not well understood. To better understand interactions of *Klebsiella pneumoniae* in response to eugenol and explore molecular regulations, transcriptome sequencing was performed. A total of 5779 differentially expressed genes (DEGs) enriched in a variety of biological processes and pathways were identified. The transcriptional data was validated by qPCR and the results showed that the expression profiles of 4 major genes involved in autoinducers-2 (AI-2) synthesis, including *luxS*, *pfs*, and *lsrK* were consistent with transcriptome analysis except for *lsrR*, a transcriptional repressor gene of lsr operon, which may repress the expression of following genes responsible for AI-2 signal transmission *in vivo*. *In vitro* AI-2 synthesis assay also revealed that eugenol could inhibit AI-2 generation. The results of our study offer insights into the mechanisms of QS inhibitory activity and *K. pneumoniae* AI-2 alterations after eugenol treatment.

## Introduction

*Klebsiella pneumoniae* (*K. pneumoniae*) is a member of Enterobacteriaceae that is widely distributed on the skin, and digestive and respiratory tracts of both humans and animals. In nature, although *K. pneumoniae* is an opportunistic pathogen, it can cause pneumonia, sepsis, and inflammation of the urinary tract of human beings and has been recognized as one of the most common community-acquired pulmonary pathogen (Podschun and Ullmann, 1998; Fung et al., 2000; Sahly et al., 2000; Ko et al., 2002). In the near decades, with widespread use of antibiotics, the incidence of multi-drug resistant and hyper virulent *K. pneumoniae* isolates has increased frequently and globally (Shon et al., 2013; Shin and Ko, 2014; Lee et al., 2016; Zhang et al., 2017). In veterinary field, *K. pneumoniae* has been thought to be less harmful to animal production for a long time. However, the emergence of newly reported multi-drug resistant and hyper virulent *K. pneumoniae* related animal infections have increased the importance of prevention and control.

Quorum sensing is an intercellular communication mechanism that regulates certain gene expressions of most bacteria by producing extracellular signaling molecules. The regulation of QS was reported to be cell-density dependent which was first found in the marine luminous bacteria *Vibrio fischeri* and *Vibrio harveyi*. In Gram-negative bacteria, the autoinducers-1 (AI-1) have been mostly identified as derivates of N-acyl homoserine lactones (AHL) which is responsible for intraspecies communication, while autoinducers-2 (AI-2), found in both Gram-negative and Gram-positive bacteria, were reported to be in charge of interspecies communication with a common chemical component of furanosyl borate diester (Diggle et al., 2007; Antunes et al., 2010; Castillo-Juarez et al., 2015). It is generally accepted that the production of AI-2 depends on LuxS encoded by the *luxS* gene (Lu et al., 2017; Park et al., 2017). It has been shown that bacterial communication mediated by LuxS is also involved in biofilm formation and lipopolysaccharide synthesis (Schauder et al., 2001; De Keersmaecker et al., 2006). Previous studies have elucidated that the substrate S-ribosylhomocysteine (SAM), a methyl donor in vital cellular processes, can yield the toxic metabolic intermediate S-adenosylhomocysteine (SAH) under catalysis of S-adenosylmethionine synthetase. The S-adenosylhomocysteine nucleosidase (Pfs) rapidly hydrolyzes SAH to adenine and S-ribosylhomocysteine (SRH). Then, SRH is converted to 4,5-dihydroxy-2,3-pentanedione (DPD) and homocysteine by LuxS. However, DPD is unstable and it has been proposed that it spontaneously rearranges to form a furanosyl borate diester, namely AI-2 (Ascenso et al., 2011). Several reports have shown the involvement of AI-2 in the regulation of expression of virulence-related factors, motility, secretion systems, and regulatory proteins (Stroeher et al., 2003).

**FIGURE 1:**
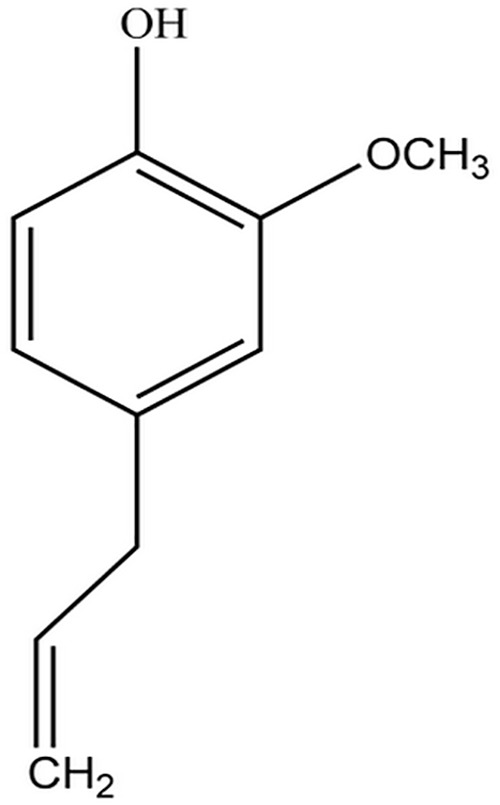
Chemical structure of eugenol.

Eugenol (Figure 1) was reported to inhibit biofilm formation at early stage and reduce preformed biofilm of *Streptococcus mutans* without affecting bacterial viability (Adil et al., 2014). Another study showed that eugenol exhibited anti-virulence properties by competitively binding to quorum sensing receptors (Rathinam et al., 2017). Moreover, it can down-regulate the expression of *S. mutans* virulence genes involved in the adhesion. Eugenol was also reported to inhibit formation of biofilm *las* and *pqs* QS systems of *Escherichia coli* biosensors at sub-MIC level (Zhou et al., 2013). *K. pneumoniae* possesses *pfs* like (*mntn*) and *luxS* orthologs in genome and can synthesize functional AI-2 molecules, so it can be inferred that group behaviors are regulated by the AI-2-mediated quorum sensing system (Balestrino et al., 2005). Despite possessing QS inhibitor activity, there was no report of AI-2 alterations at transcription levels and molecular regulation of quorum sensing in *K. pneumoniae* after treated with eugenol at sub-MIC.

Therefore, the current study analyzed transcriptional changes and interactions of AI-2 of clinical *K. pneumoniae* isolate treated with eugenol at exponential phase, and aimed to provide a better understanding of the QS inhibitory mechanism of eugenol.

## Materials and Methods

### Chemicals, Bacteria, and Culture Medium

The eugenol used in this study was obtained from Macklin Biochemical (Shanghai, China) Co., Ltd. Dimethyl sulfoxide (DMSO, Sigma-Aldrich, United States) was added with a final concentration of 1%. Triphenyltetrazolium chloride (TTC) was purchased from Biotopped Co., LTD (Beijing, China). *K. pneumoniae* ATCC 46117 and *E. coli* ATCC 25922 were kept in our laboratory. The clinical *K. pneumoniae* was isolated from nasal swabs and the feces sample of a porcine with respiratory disease was collected from a small pig farm in Gongzhuling City, Jilin province. Tryptone soybean broth (TSB) and Tryptone soybean agar (TSA) were obtained from Qingdao Hope Bio-Technology Co., LTD (Qingdao, China). Commercial antibiotic paper disks: AMO (Amoxicillin, 20 μg), AZM (Azithromycin, 15 μg), AZL (Azlocillin, 75 μg), AZT (Aztreonam, 30 μg), CFZ (Cefazolin, 30 μg), CHL (Chloramphenicol, 30 μg), CTX (Cefotaxime, 30 μg), DOX (Doxycycline, 30 μg), FRZ (furazolidone, 300 μg), GEN (gentamicin, 10 μg), IPM (imipenem, 10 μg), KAN (kanamycin, 30 μg), LVX (levofloxacin, 5 μg), OXA (oxacillin, 1 μg), NOR (Norfloxacin, 10 μg), POL (PolymyxinB, 300IU), STR (Streptomycin, 10 μg) were purchased from Hangzhou Microbial Reagent Co., Ltd (Hangzhou, China). S-adenosylhomocysteine (SAH) and DTNB were purchased from Sigma-Aldrich, Inc. (United States).

### Drug Susceptibility and Antibacterial Test

The drug susceptibility of the clinical isolated *K. pneumoniae* was determined using agar disk diffusion method. Briefly, bacterial isolate was cultured in TSB at 37°C to reach a turbidity of 0.05 at 600 nm (approximately 10^8^ cfu/mL) and was further diluted to obtain 10^6^ cfu/mL. Sterile plates containing TSA medium were seeded with 100 μl each bacterial strain. Seventeen kinds of commercial antibiotic paper disks were placed on the surface of the inoculated plates. Incubations were carried out for 16 h at 37°C. Antibacterial susceptibility was determined by measuring the diameter of the inhibition zone (in mm) generated around the disc according to the manufacturer’s protocol. Discs of cefazolin were used as positive controls. All tests were performed in triplicate. *K. pneumoniae* ATCC 46117 and *E. coli* ATCC 25922 were employed in this study as quality control strains. For MIC determination, the eugenol was serially diluted and inoculated with an equal volume of bacterial suspension to make final concentrations of 8, 4, 2, 1, 0.5 μg/mL. 1% TTC was added as growth indicator. The tubes containing TSB only and TSB with bacteria were set as negative and positive controls, respectively. MIC value was defined as the lowest concentration of the samples that resulted in complete inhibition of visible growth after incubation. Each test was repeated triplicate at separate times.

### Samples Treatment and Collection

The *K. pneumoniae* isolate was cultured to reach a turbidity of 0.05 at 600 nm. Then, eugenol was added into tube to reach a final concentration of sub-MIC (0.5 μg/mL) for a further culture of 1 h. In the control, an equal volume of TSB was added instead of eugenol. 2 mL bacterial culture was centrifuged at 8000*g*, for 5 min for RNA extraction. In each group, triplicate samples were prepared under the same experimental conditions.

### RNA Extraction, Library Construction, and Transcriptome Sequencing

The library construction and sequencing were performed at Biomarker Technology (Beijing, China) using Illumina HiSeq 2500 platform. Total RNA extraction of bacterial samples was performed using a RNAiso Pure RNA Isolation Kit (TaKaRa, JP) followed by DNase I digestion, carried out according to the manufacturer’s instruction. The quality and quantity of RNA was assessed using a Thermo Scientific NanoDrop 2000 UV-Vis Spectrophotometer (Thermo Fisher Scientific, United States), a Qubit 2.0 fluorometer (Life Technologies, United States) and an Agilent 2100 Bioanalyzer system (Agilent Technologies, United States).

The prokaryotic rRNA was eliminated by complementary probe sequences. The rRNA-depleted RNA was randomly fragmented into small pieces by adding a fragmentation buffer and served as template for synthesis of the first strand of cDNA by using superScript II reverse transcriptase and random hexamers, then, the second strand of cDNA was synthesized by adding a buffer, dNTPs, dATP, dGTP, dCUP, RNaseH, and DNA polymerase I subsequently. The double-stranded cDNA was purified using AMPure XP beads and was subjected to end repair, adapter ligation, base A addition, adaptor ligation and agarose gel electrophoresis filtration. Suitable fragments was selected and enriched as templates by PCR amplification and used for cDNA library preparation. For library quality control, the cDNA library concentration and insert size was measured by using a Qubit 2.0 fluorometer and Agilent 2100 Bioanalyzer system, and effective concentration was qualified by qPCR.

Transcriptome analysis was done using reference genome-based reads mapping. Briefly, raw reads were pre-processed by removing adaptor, primer sequences and ambiguous reads. Additionally, low-quality reads (including reads with more than 10% unknown nucleotides and reads with the percentage of the bases with quality scores Q20 lower than 50%) were filtered before alignment. Hereafter, high quality clean reads were mapped with reference *K. pneumoniae* strain Kp52.145 sequence (GenBank: FO834906.1) by Bowtie2 software to obtain position and characteristic information. RNA-seq data were further analyzed and transcript expression levels were determined by calculating FPKM (Fragments Per Kilobase of transcript per Million mapped reads) by Cuffdiff section of Cufinks software.

### Differential Expression Gene Analysis

The control (CK1) and eugenol treated groups (EK1) were subjected to differential gene expression analysis using Empirical Analysis of Digital Gene Expression Data (edgeSeq) R package version 3.22.3 to identify DEGs. For comparison, the *P*-values calibrated for multiple tests were performed according to the Benjamini and Hochberg method. False discovery rate (FDR) threshold ≤0.05 and | log_2_fold change (FC)| >1 were set as the criteria to determine significant DEGs (Sun et al., 2015).

### GO and KEGG Enrichment Analysis

Gene Ontology (GO) enrichment analysis and KEGG (Kyoto Encyclopedia of Genes and Genome) pathway analysis were performed based on selected DEGs (Sun et al., 2017; Wang et al., 2018). Briefly, DEG was mapped to terms in GO database and the gene number of each term was counted to analyze the GO and KEGG pathway enrichment. The GO terms and pathway with corrected *P*-value by FDR, *Q*-value ≤ 0.05 are considered significantly enriched. The major biological functions that DEGs perform can be evaluated by analysis of significant GO and KEGG pathway enrichment.

### qPCR

Expression of *luxS*, *pfs*(*mtnn*), *lsrR*, and *lsrK* genes at mRNA level was evaluated by qPCR. Bacteria was cultured to reach turbidity of 0.05 at 600 nm, eugenol was added to make a final concentration of 1/4 MIC. In the control group, TSB was added instead of eugenol. The mixture was incubated at 37°C and shaken at 150 rpm for 1 h. Total RNA was extracted using RNAiso Plus (TaKaRa) and was reversely transcribed to cDNA by PrimeScript RTase. Then, the cDNA was used as template for Real Time PCR amplification in the same reaction tube using One Step SYBR^®^PrimeScript^TM^ RT-PCR Kit (TaKaRa).

Real-time PCR process was performed on Applied Biosystems 7500 Real-Time PCR System (United States). The primers (Table 1) were designed according to a previous study and *K. pneumoniae* str.52.145 complete genome sequence. The amplifications were performed in 20 μl reaction mixtures containing 10 μl One Step SYBR RT-PCR Buffer Ø, 0.4 μl TaKaRa Ex Taq HS, 0.4 μl PrimeScript RT Enzyme Mix Π, 0.4 μl ROX Reference Dye Π, 6.0 μl RNase Free H_2_O, 0.4 μl forward and reverse primer (10 μM), respectively, and 2.0 μl template. 16S rRNA was used as reference gene (Table 1). The reacting condition was set as a two steps method as follows: 1 cycle for reverse transcription at 42°C, 5 min and 95°C for 10 s, then, 40 cycles consisting of denaturation at 95°C for 5s and annealing at 60°C for 34 s. All templates were run in triplicate.

**Table 1:** Sequences of primers used in this study.

Primer name	Sequence (5′-3′)	Product size (bp)
*luxs*-F	ACGCCATTACCGTTAAGATG	81 (Han et al., 2013)
*luxs*-R	AGTGATGCCAGAAAGAGGGA	
*pfs*-F	CGGCAACAGCCAGGAACTCA	169 (Han et al., 2013)
*pfs*-R	GCGAAAATCCGCCACAACTT	
*lsrR*-F	AGATCCATCGGGAACTGC	115
*lsrR*-R	CCAATCAGAGCGGCATAA	
*lsrK*-F	GCATTCGGGCGGTTATTT	124
*lsrK*-R	TGCCAGTTGGTGGTAAGG	
16SrRNA-F	TGTCGTCAGCTCGTGTTGTG	130
16SrRNA-R	ATCCCCACCTTCCTCCAGTT	

### Enzyme Activity Inhibition Assay

*In vitro* synthesis of AI-2 was performed on a 96-well plate according to the method described before with little modification (Ascenso et al., 2011). Briefly, LuxS and Pfs (MtnN) were pre-prepared by prokaryotic expression previously and both were purified by AKTA pure system (GE, United States). SAH was incubated with 100 μl LuxS and Pfs to obtain an initial concentration of 1 mg/mL each in PBS (pH 7.5). Eugenol was added to the mixture with final concentrations of 0.5, 1, and 2 μg/mL, respectively. For control, equal volume of PBS was inoculated with the same experimental condition instead. The plate was incubated at 37°C for 1 h and then ultrafiltered to removing extra reaction protein. Then, 100 μl filtrate, with the major component of homocysteine, was added to 5 mmol/L equal volume Ellman’s reagent (DTNB, dissolved in 100 mmol/L PBS and 0.1 mmol/L EDTA, pH 7.2) and incubated at 37°C for 15 min. The absorbance of obtained supernatant was measured by Thermo Fisher Scientific NanoDrop 2000 UV-Vis Spectrophotometer (Thermo Fisher Scientific, United States) at 412 nm.

## Results

### Drug Susceptibility Test

As shown in Table 2, the clinical isolated porcine-derived *K. pneumoniae* was resistant to three out of seventeen kinds of commonly used antibiotics: amoxicillin, azithromycin, and oxacillin. The result suggested that the *K. pneumoniae* isolate exhibited a multi-drug resistant phenotype and is mainly resistant to beta-lactam antibiotics. The MIC of eugenol against *K. pneumoniae* isolate was 2 μg/ml.

**Table 2:** Antimicrobial resistance profile of clinical *K. pneumoniae* isolate.

Antibiotics used	Antimicrobial resistance^∗^
AMO	R
AZM	S
AZL	R
AZT	S
CFZ	S
CHL	S
CTX	S
DOX	S
FRZ	I
GEN	S
IPM	S
KAN	S
LVX	S
OXA	R
NOR	S
POL	S
STR	S

*^∗^R, resistant; I, intermediate; S, sensitive.*

### Transcriptome Sequencing and Clustering DEGs

**FIGURE 2:**
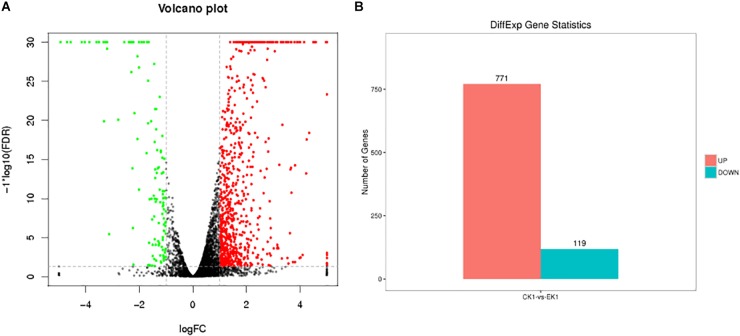
Volcano graph of DEGs **(A)** and up-regulated and down-regulated genes **(B)** between eugenol treated group (EK1) and control group (CK1).

Raw reads data were sequenced and filtered for further analysis, yielding approximately 14.7–20.1 million clean reads. The number of high-quality clean reads in both the eugenol treated group and control group is over 98.5%. The rate of low-quality reads is lower than 0.7%. Statistical analysis revealed high total number of reads of sequencing samples and high ratio of high-quality reads. The results suggested good quality sequencing data. After aligning the high-quality clean reads with the rRNA, the reads were mapped to a reference genome. Transcriptome sequencing data were deposited to NCBI SRA database (SRA accession: PRJNA504310). In total, 5779 reference genes were detected, with a known gene number of 4514 (78.11%) in control group and 4561 (78.92%) in the eugenol treated group. Based on FDR threshold ≤0.05 and | log2 fold change (FC)| >1, 890 significant DEGs were identified. Among which, 771 genes were up-regulated while the rest 119 genes were down-regulated in control group vs. eugenol treated group (Figure 2).

### Functional Annotation and Pathway Analysis of DEGs

**FIGURE 3:**
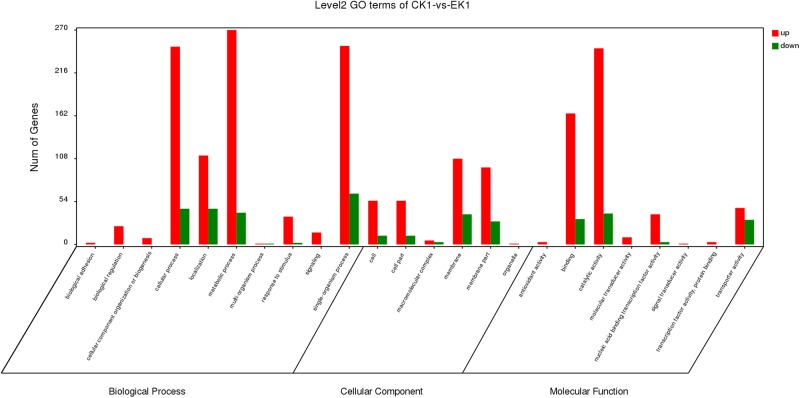
DEGs of biological process, cellular component and molecular function GO terms.

Gene Ontology analysis can not only provide reliable gene product descriptions from various databases but also offers a set of dynamic, controlled, and structured terminologies to describe gene functions and products in organism. According to GO functions, all DEGs were classified into three categories: biological process, cellular component, and molecular function (Figure 3). There were a total of 77 terms enriched in GO terms (eugenol treated group vs. control group) among which 39 for biological process, 6 for cellular component and 32 for molecular function (Supplementary Figures S1 A–C). The most represented categories were “membrane” (GO:0016020), “intrinsic component of membrane” (GO:0031224), “membrane part” (GO:0044425), “oxidoreductase activity” (GO:0016491), “transporter activity” (GO:0005215), “transmembrane transporter activity” (GO:0022857), “tetrapyrrole binding” (GO:0046906), “establishment of localization” (GO:0051234), “localization” (GO:0051179) and “single-organism process” (GO:0044699). Among which, 146 out of 604 genes in “membrane,” 126 out of 529 genes in “intrinsic component of membrane,” 126 out of 536 genes in “membrane part,” 63 out of 228 genes in “oxidoreductase activity,” 77 out of 314 genes in “transporter activity,” 68 out of 270 genes in “transmembrane transporter activity,” 7 out of 9 genes in “tetrapyrrole binding” 157 out of 642 genes in “localization,” and 157 out of 640 genes in “establishment of localization” were up-regulated in control vs. eugenol treated groups. Results showed that gene expression of *K. pneumoniae* after eugenol treatment was up-regulated or down-regulated compared with untreated cells indicating molecular interactions to different gene ontology.

### KEGG Pathway Analysis

**FIGURE 4:**
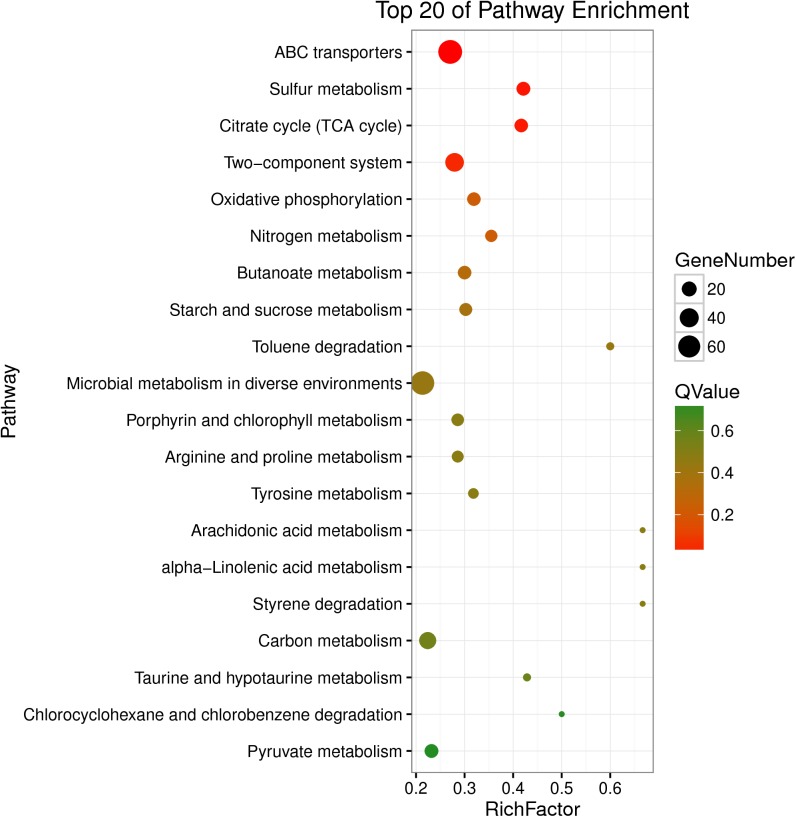
Statistical enrichment of DEGs in KEGG pathways.

The DEGs were also enriched in KEGG pathways, which could provide data on biological systems and their relationships at molecular, cellular, and organism levels. The KEGG pathways were annotated from the assembled *K. pneumoniae* transcriptome, and results were mapped with GO terms. As shown in Figure 4, among the top 20 of pathway enrichment, there were 60 DEGs belonging to ABC transporters and microbial metabolism in diverse environments pathways each. 40 DEGs were secondly enriched in two-component system pathway which in charge of signal transduction. Moreover, citrate cycle (TCA cycle), sulfur metabolism, ABC transporters and two-component system were the most significantly enriched pathways at both *P*-value and *Q*-value level (<0.05). The rest of DEGs, mainly enriched in metabolic pathways, were up-regulated (Supplementary Figures S2 A–C). The results indicated that eugenol affected bacterial metabolic pathways which may further inhibit AI-2 molecular production and biofilm formation.

### Gene Expression Profile Analysis

**FIGURE 5:**
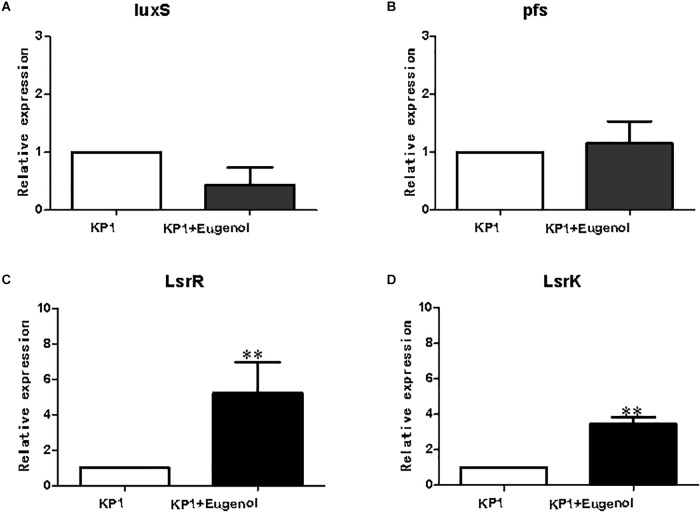
Relative expression of AI-2 relevant genes. luxS **(A)**, pfs **(B)**, lsrR **(C)**, lsrK **(D)**. All data were expressed as mean ± SD., *n* = 3. ^∗∗^*P* < 0.01 vs. control group.

qPCR analysis was performed to validate the expression profiles of DEGs in bacteria. As shown in Figure 5, genes involved in AI-2 production were selected as targets for qPCR analysis. The qPCR results of *luxS*, *pfs*(*mtnn*), and *lsrK* agreed with the fpkm dataset. In contrast, the relative expression of *lsrR* in eugenol added group was significantly higher than non-eugenol group despite very little difference between fpkms. Although the fold changes in *K. pneumoniae* slightly differed between qPCR and RNA-seq, the general expression trends matched.

### AI-2 Synthesis Inhibition Test

**FIGURE 6:**
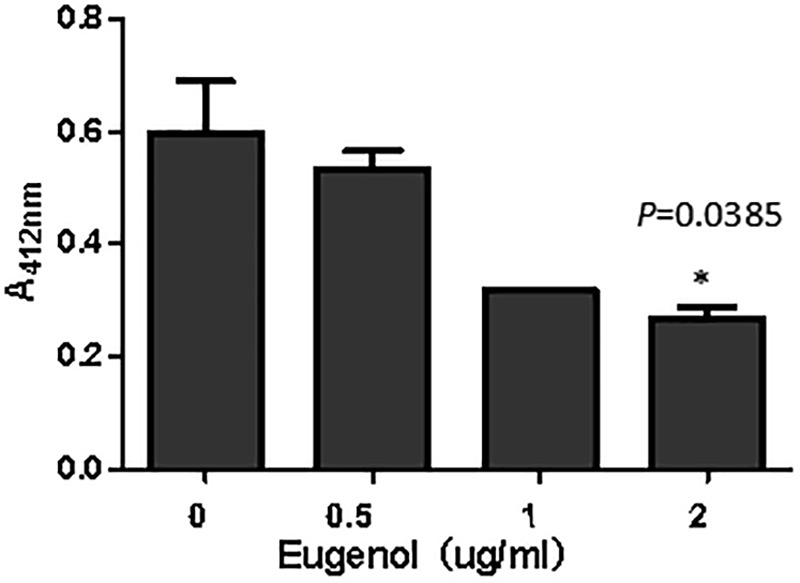
Absorption of homocysteine incubated with different concentrations eugenol. ^∗^*P* < 0.05 vs. control group.

The synthesis of AI-2 *in vitro* can be evaluated via measurement of homocysteine that is generated with AI-2 simultaneously. As can be seen from Figure 6, addition of eugenol at MIC could significantly (*P* = 0.0385) inhibit the production of AI-2 molecule compared with the control group. The absorbance of homocysteine decreased after treatment with eugenol at 1/2 MIC and 1/4 MIC, despite the non-significant difference, indicating lower AI-2 levels. The above result clearly demonstrated that eugenol could interact with LuxS and Pfs (MtnN) and thus interfere the generation of AI-2. The effect presented a dose-dependent manner. Furthermore, the result was in accordance with transcriptome sequencing and qPCR analysis, which further indicated the inhibiting effect of eugenol on *K. pneumoniae* AI-2 molecule and quorum sensing system.

## Discussion

Animal-derived multi-drug resistant *K. pneumoniae* has been reported more frequently in recent years. The emergence of animal infection caused by *K. pneumoniae* not only results in economic loss of animal husbandry, but will pose a great challenge to human public health. However, due to the wide use of antibiotics, fast occurring bacterial drug-resistance has made most conventional antibiotics invalid, which make the situation more severe (Zou et al., 2011; Overdevest et al., 2014; Vuotto et al., 2014, 2017; Anes et al., 2017). According to drug-resistance result, the isolated porcine-derived *K. pneumoniae* was multi-drug resistant to β-lactamase antibiotics, which was commonly found in both human and animal clinical trials. As an effective herbal monomer, eugenol was proven to possess QS inhibitor activity. However, little is known about how *K. pneumoniae* AI-2 responses to eugenol at the transcriptional level. The present study revealed that eugenol could decrease AI-2 signal molecules mainly by down-regulating *luxS* gene expression, up-regulating negative feedback gene *lsrR*, and inhibit catalytic activity of LuxS.

Quorum sensing consists of an enzyme that catalyzes the synthesis of the signal molecules and receptors that bind to the signal and reprograms the expression of several genes. Previous study has investigated the theory that AI-1 was not produced by *K. pneumoniae* due to the AI-1 type QS quenching enzymes, AHL lactonases (Park et al., 2003; Sun et al., 2016). In contrast, class II autoinducer, AI-2 could be detected in culture supernatant and was LuxS dependent (Wang et al., 2005; Ascenso et al., 2011; Zhu et al., 2011). Moreover, it is well accepted that QS can affect biofilm formation and virulent gene expression by regulating LPS synthesis and virulent genes expression, both of which are closely related to drug-resistance (De Araujo et al., 2010; Defoirdt, 2018). Therefore, on the basis of the above study, the transcriptional analysis was performed and validated by qPCR. Interactions between eugenol and AI-2 were confirmed by AI-2 inhibition test.

**FIGURE 7:**
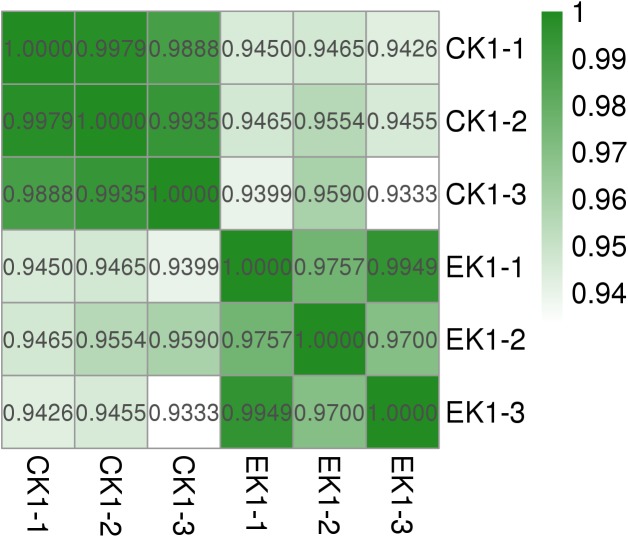
Pearson’s correlation coefficient and volcano graph of eugenol treated group (EK1) and control group (CK1).

**FIGURE 8:**
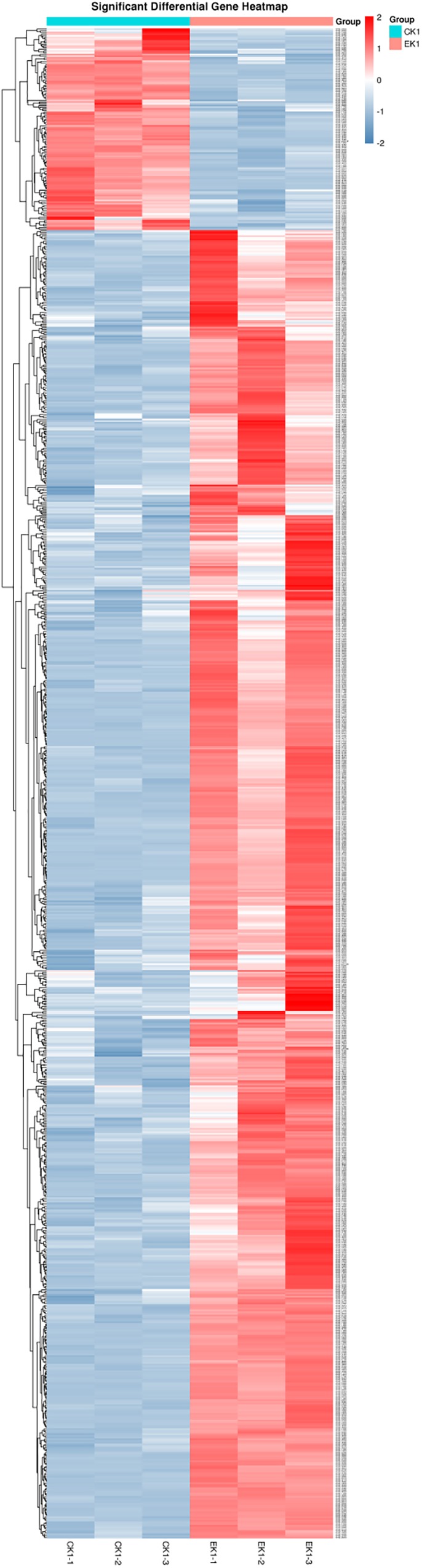
Heatmap expression pattern clustering analysis of eugenol treated group (EK1) and control group (CK1).

Concerning the results of transcriptome sequencing, the total number of reads and ratio of high quality was high, and the error rate was low, suggesting good quality sequencing data. The correlation between control and eugenol treated groups was assessed using Pearson’s correlation coefficient (Figure 7). The paired Pearson’s correlation co-efficient within groups were all higher than 0.97, demonstrating good sample correlations. The heat maps (Figure 8) of the induced and suppressed transcripts of eugenol-exposed samples matched to controls in the opposite way. This result demonstrated that exposure to eugenol involved a series of gene ontologies mainly in biological processes, cellular component and molecular function, and could consequently result in the up-regulating most genes. Moreover, the result also explained the eugenol mode of action on bacteria at sub-MIC.

*lsrK* and *lsrR* are positive and negative regulating genes of *luxS*, respectively. *lsrK* can positively regulate phospho-AI-2 production and negatively regulate *lsrR*. *lsrR*, inversely, not only negatively regulate subsequent gene cluster from *lsrA* to *lsrG*, but *lsrK* and itself (Taga et al., 2003; Torres-Escobar et al., 2013; Adams et al., 2014). In the study, the fpkms of *luxS* and *lsrR* were a little lower in the eugenol treated group vs. control, while *mtnN* and *lsrk* were higher. For *luxS* and *pfs*, it could be explained that although expression of *luxS* and *pfs* was coincidence at the initial culture period, the speed and level really differed when bacterial growth reach logarithmic phase, which has been proven in previous research. The results of qPCR were consistent with *luxS*, *pfs*(*mtnN*), and *lsrK* except for *lsrR*, a transcriptional repressor of lsr operon. In the eugenol treated group, the relative expression of *lsrR* was significantly higher than that of the control. Concerning the non-significant fpkm data between control and eugenol-treated group, we speculate that the high level of *lsrR* does repress the expression of the following genes responsible for AI-2 signal transmission, which will reduce the level of phospho-AI-2 *in vivo*. The multidrug resistance related proteins (MRPs) function as efflux transporters of a variety of large organic anions or their conjugates. Eugenol was reported to decrease MRP2 levels more effectively than Piper betel leaves extract with less of a sensitizing effect and therefore has the potential to serve as an additive to reduce drug resistance (Young et al., 2006). ATP-binding cassette (ABC) transporters are membrane-bound transporters involved in various physiological processes. Previous study found that ABC transporter encoding genes MgAtr1, MgAtr2, MgAtr4, and MgAtr5 from the wheat pathogen *Mycosphaerella graminicola* could be upregulated. Although the mode of action of eugenol is unclear, there is some evidence that it might act as an uncoupler of oxidative phosphorylation and thus can inhibit ABC transporter activity by depleting ATP. Moreover, these findings also support the hypothesis that the encoded ABC transporters provide protection against toxic compounds present in the environment of the fungus (Zwiers and De Waard, 2000; Stergiopoulos et al., 2002).

*In vitro* synthesis of AI-2 has been applied to detect and qualify the AI-2 of Avian Pathogenic *E. coli* before (Han et al., 2013). In this study, the addition of eugenol at MIC significantly inhibited the generation of AI-2 molecule, and 1/2 MIC and 1/4 MIC also exhibited inhibiting effects. The above results clearly demonstrated that eugenol could interact with *luxS* and *mtnN* and thus interfere with the generation of AI-2, and the effect was dose-dependent. Combined with transcriptome sequencing and qPCR analysis, we think sub-MIC eugenol exerted AI-2 inhibitory activity mainly depends on *luxS* and AI-2 synthesis regulator gene affiliated to biological process and molecular function ontology.

## Conclusion

In conclusion, the study revealed the transcriptional profile of clinical isolated porcine-derived *K. pneumoniae* treated with sub-MIC eugenol. The DEGs involved in AI-2 molecules were identified. Eugenol could reduce AI-2 molecule generation largely by lowering AI-2 synthesis gene category e.g., *luxS* and *lsrK*, increasing the expression of repressive gene *lsrR*, and decelerating LuxS catalytic activity. The research provides a basis for elucidating the mechanism of interactions between eugenol and *K. pneumoniae*.

## Author Contributions

Y-MW contributed to drafting and revising the manuscript. Y-MW, L-CK, and W-LD participated in the design of the study and data analysis. HX-M participated in experiment design and article revise, and provided constructive advice to the study. Y-MW and KO performed the antimicrobial susceptibility test, qPCR, and enzyme activity inhibition assay. All authors read and approved the final manuscript.

## Conflict of Interest Statement

The authors declare that the research was conducted in the absence of any commercial or financial relationships that could be construed as a potential conflict of interest.

## Supplementary Material

FIGURE S1 GO enrichment analysis of *P*-value heat map **(A)**, *Q*-value heat map **(B)**, and enrichment factor heat map **(C)**.

FIGURE S2 KEGG enrichment analysis of *P*-value heat map **(A)**, *Q*-value heat map **(B)**, and enrichment factor heat map **(C)**.
