# Supplementary figures and images for: Transcriptome Analysis Reveals AI-2 Relevant Genes of Multi-Drug Resistant Klebsiella pneumoniae in Response to Eugenol at Sub-MIC
Source: Front Microbiol. 2019 May 28;10:1159. doi: 10.3389/fmicb.2019.01159 (PMC6547871; doi:10.3389/fmicb.2019.01159)

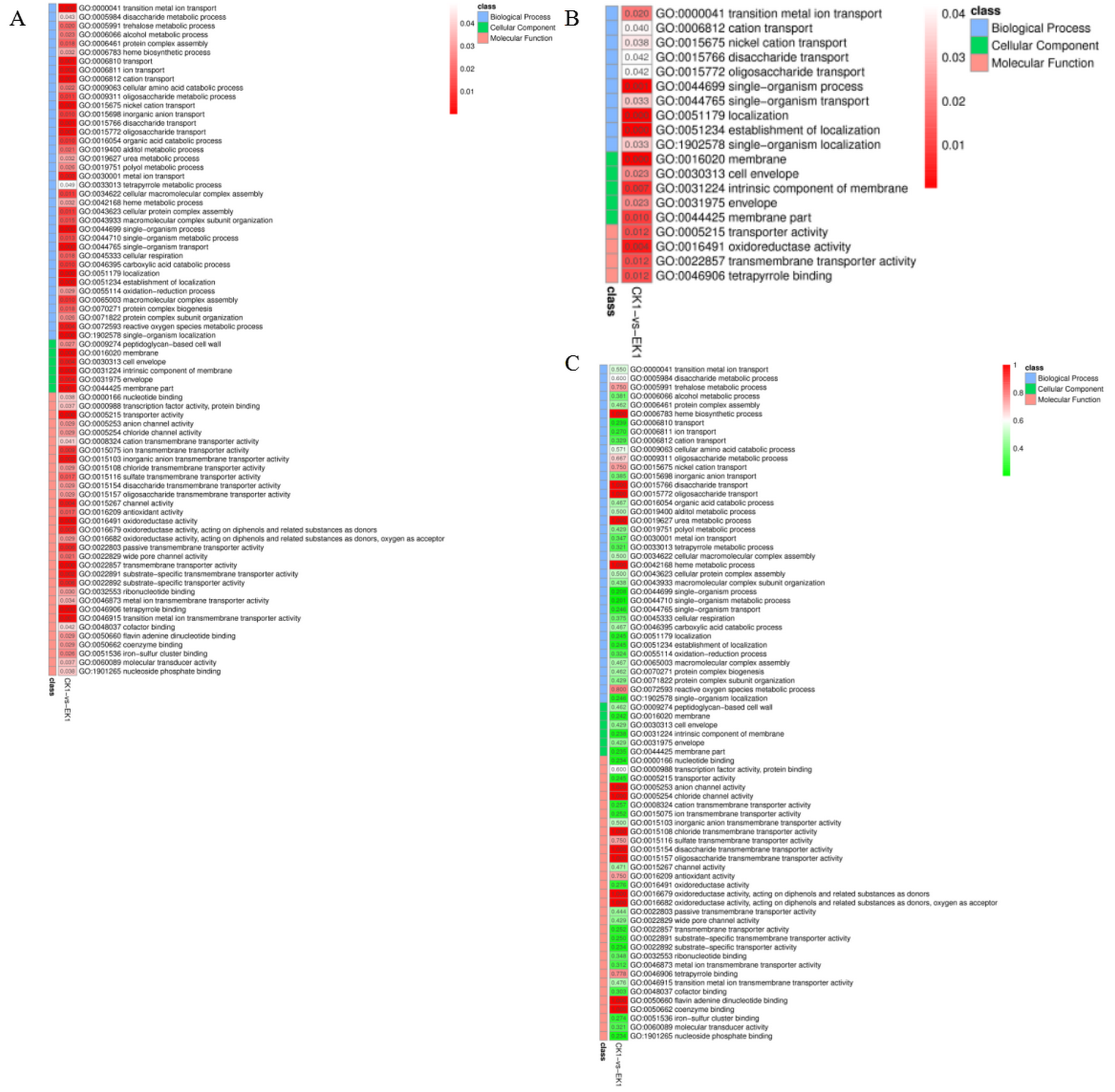

Supplement: FIGURE S1 — GO enrichment analysis of P-value heat map (A), Q-value heat map (B), and enrichment factor heat map (C). [file Image_1.TIF]

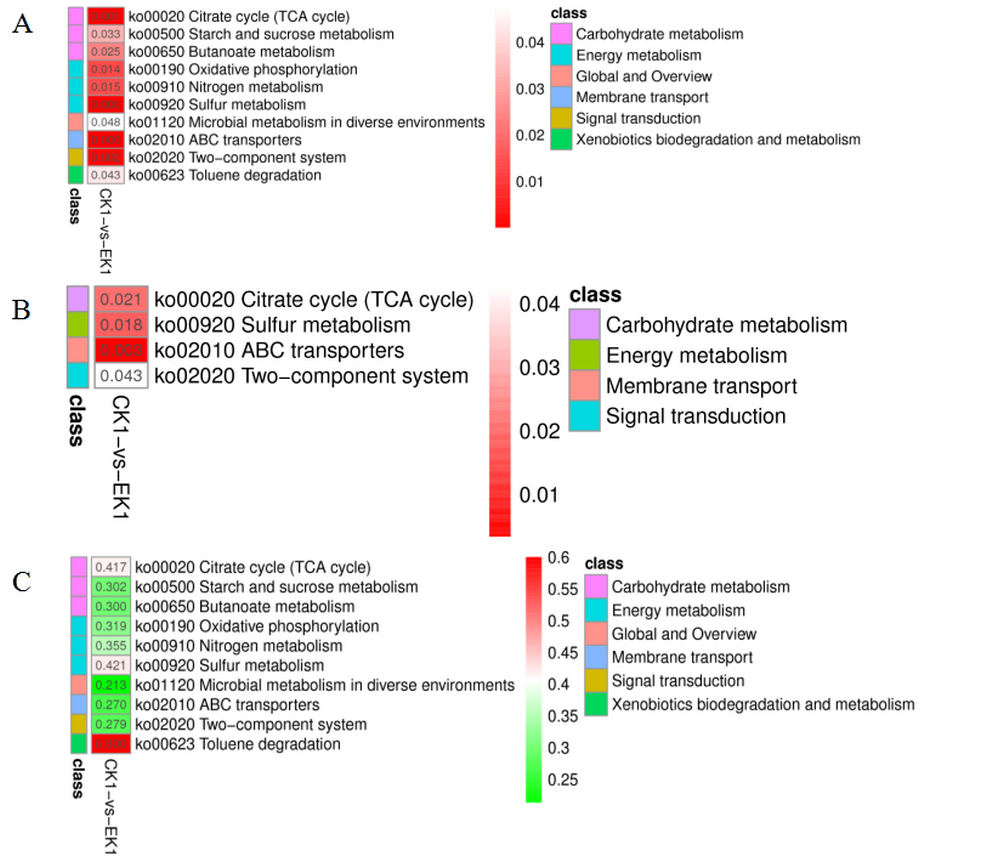

Supplement: FIGURE S2 — KEGG enrichment analysis of P-value heat map (A), Q-value heat map (B), and enrichment factor heat map (C). [file Image_2.TIF]
